# The Transdiagnostic Self-Injury Interview: A Feasibility Study

**DOI:** 10.1192/j.eurpsy.2022.1522

**Published:** 2022-09-01

**Authors:** J.N. Kjaer, T. Holm, V. Bliksted, T. Ellegaard, S. Dolmer, O. Mors

**Affiliations:** Aarhus University Hospital, Dept. For Psychosis, Skejby, Denmark

**Keywords:** self-injury, Transdiagnostic, Validation study, Psychosis

## Abstract

**Introduction:**

Non-suicidal self-injury (NSSI) is associated with emotional distress and mental disorders. In clinical samples NSSI is reported by 21% to 60% of all psychiatric patients. Developed NSSI instruments are not suitable for clinical settings because they are too time-consuming or lack validation across psychiatric diagnoses.

The Transdiagnostic Self-Injury Interview (TSI) is semi-structured interview that accesses onset, frequency, methods, and severity of NSSI. It is transdiagnostic and developed for clinical settings.

**Objectives:**

The purpose of the study is to evaluate the feasibility of a TSI validation study. The study will also provide preliminary validation of the instrument.

**Methods:**

The feasibility study will recruit participants at in- and outpatient units from a university hospital. Participants can be included in the study if they are 18 years old and admitted to a psychiatric in- or outpatient unit.

Instruments: The Deliberate Self-Harm Inventory will be used to test concurrent validity. Convergent validity will be tested with the Columbia Suicidality Severity Rating Scale, the Personal and Social Performance scale, the Affective Lability Scale-short, and the Brief Trauma Questionnaire. Interrater reliability will be evaluated in groups of medical doctors, psychologist, and other clinical professionals.

Feasibility are measured by inclusion of participants per week, the time each participant takes to complete the study instruments, and number of dropouts.

**Results:**

Recruitment of participants will start in the fall of 2021. We aim to recruit 50 participants.

**Conclusions:**

When TSI has been validated, it can be used to assess prevalence and severity of NSSI and clarify the need for treatment and supervision.

**Disclosure:**

No significant relationships.

